# German specialists treating testicular cancer follow different guidelines with resulting inconsistency in assessment of retroperitoneal lymph-node metastasis: clinical implications and possible corrective measures

**DOI:** 10.1007/s00345-023-04364-5

**Published:** 2023-04-04

**Authors:** Justine Schoch, Kathrin Haunschild, Angelina Strauch, Kai Nestler, Hans Schmelz, Pia Paffenholz, David Pfister, Thorsten Persigehl, Axel Heidenreich, Tim Nestler

**Affiliations:** 1 Department of Urology, Federal Armed Forces Hospital Koblenz, Ruebenacherstrasse 170, 56072 Koblenz, Germany; 2 Department of Urology, Faculty of Medicine, University Hospital Cologne, University Cologne, Cologne, Germany; 3 Institute of Diagnostic and Interventional Radiology, Federal Armed Forces Hospital Koblenz, Koblenz, Germany; 4 Institute for Diagnostic and Interventional Radiology, Faculty of Medicine, University Hospital Cologne, University Cologne, Cologne, Germany; 5 Department of Urology, Medical University Vienna, Vienna, Austria

**Keywords:** Germ cell tumor, Guideline adherence, Testicular cancer, Retroperitoneal lymph-node metastasis, RECIST 1.1, Imaging

## Abstract

**Background:**

Testicular germ cell tumors (GCTs) are aggressive but highly curable tumors. To avoid over/undertreatment, reliable clinical staging of retroperitoneal lymph-node metastasis is necessary. Current clinical guidelines, in their different versions, lack specific recommendations on how to measure lymph-node metastasis.

**Objective:**

We aimed to assess the practice patterns of German institutions frequently treating testicular cancer for measuring retroperitoneal lymph-node size.

**Methods:**

An 8‐item survey was distributed among German university hospitals and members of the German Testicular Cancer Study Group.

**Results:**

In the group of urologists, 54.7% assessed retroperitoneal lymph nodes depending on their short-axis diameter (SAD) (33.3% in any plane, 21.4% in the axial plane), while 45.3% used long-axis diameter (LAD) for the assessment (42.9% in any plane, 2.4% in the axial plane). Moreover, the oncologists mainly assessed lymph-node size based on the SAD (71.4%). Specifically, 42.9% of oncologists assessed the SAD in any plane, while 28.5% measured this dimension in the axial plane. Only 28.6% of oncologists considered the LAD (14.3% in any plane, 14.3% in the axial plane). None of the oncologists and 11.9% of the urologists (*n* = 5) always performed an MRI for the initial assessment, while for follow-up imaging, the use increased to 36.5% of oncologists and 31% of urologists. Furthermore, only 17% of the urologists, and no oncologists, calculated lymph-node volume in their assessment (*p* = 0.224).

**Conclusion:**

Clear and consistent measurement instructions are urgently needed to be present in all guidelines across different specialistic fields involved in testicular cancer management.

**Supplementary Information:**

The online version contains supplementary material available at 10.1007/s00345-023-04364-5.

## Introduction

Testicular germ cell tumors (GCTs) are a mostly aggressive cancer entity with an age-adjusted incidence rate of 1.5 per 100.000 individuals worldwide. Furthermore, GCTs are the most common cancer entity occurring in young male adults [1]. The worldwide incidence of GCTs has increased over the past 30 years, while the mortality rates have decreased due to improvements in chemo- and radiotherapy [2, 3].

Survival rates mainly depend on three factors: the clinical stage, the histological findings, and the *International Germ Cell Cancer Collaborative Group* (IGCCCG) prognosis group for patients with metastasis. Survival rates vary anywhere from 95 to 100% for localized disease to 70–90% for metastasized disease depending on IGCCCG classification [4].

**Fig. 1:**
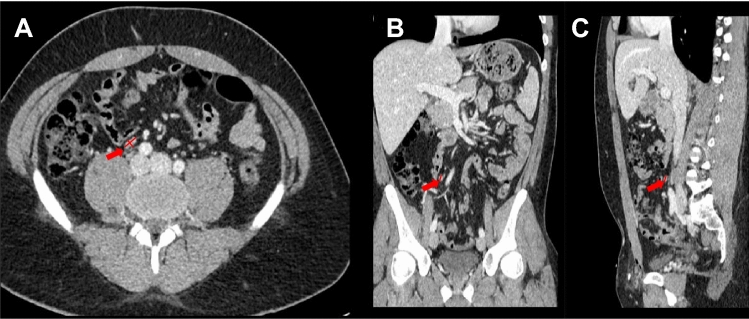
**A** 27-year-old patient with unclear retroperitoneal lymph nodes at CT staging (right-sided nonseminomatous germ cell tumors (NSGCT) (yolk sack tumor), pT1, L0, V0, R0 **A**. Unclear retroperitoneal lymph node at the critical landing zone with an LAD of 12 mm and SAD of 5 mm in the axial plane. **B** The same lymph node with an LAD of 9 mm in the coronal plane and **C** with an LAD of 9 mm in the sagittal plane. This is an example of an interpretation issue in clinical staging, as this patient could be classified as cN0 or cN1, resulting in a decision between surveillance and further therapy. This patient did not receive any further treatment and followed up imaging showed constant lymph nodes, classifying as non-metastatic cN0. CT images were kindly provided by Dr. Waldeck, Department of Radiology, Federal Armed Services Hospital Koblenz, Germany

Therapy based on the clinical stage and histology results is necessary to achieve optimal treatment results, according to the current guideline recommendations [4]. Consequently, the first step to establishing an appropriate individual therapy regimen besides the histology and the serum concentration of tumor markers is clinical staging using cross-sectional imaging to assess the presence of lymph-node (cN) and systemic metastasis (cM). Axial dimension was the only obtainable dimension for a long time after CT imaging has become a standard diagnostic. In line, this was the only dimension to measure lymph nodes and has been used as the main criteria in studies related to staging and treatment decisions. In recent years, reconstruction capacities have been developed resulting in the availability of coronal and sagittal views in addition to the axial dimension. This resulted in confusion of which radiological diameter should be considered in the assessment of lymph nodes. However, the current guidelines [European Association of Urology (EAU) [5], National Comprehensive Cancer Network (NCCN) [6], German Society of Urology (DGU) [7], European Society for Medical Oncology (ESMO) [8], *onkopedia* (the Guidelines of the Medical Societies in Hematology and Medical Oncology of the German speaking countries)] lack specific and consistent recommendations [9]. In addition, there are RECIST 1.1 criteria with definitions how to measuring lymph-node metastasis radiologically (plane, dimension), but RECIST 1.1 is not widely used in clinical routine and there can be an inconsistency in how the radiologically described measurements are clinically interpreted [6, 10, 11]. These differences could lead to different stages, as shown in Fig. 1. Similarly, the current studies suggest that magnetic resonance imaging (MRI) is similar to computed tomography (CT) regarding sensitivity and specificity, yet CT imaging is still the most common imaging method and is primarily recommended in the current guidelines [12–14].

Therefore, the aims of this study were to evaluate the following practice patterns among German specialists in treating GCTs: (1) assessment of retroperitoneal lymph nodes for primary clinical staging and the presence of a residual mass after chemotherapy; (2) use of abdominal MRI for the initial clinical staging and follow-up; and (3) GCT guidelines’ adherence.

## Materials and methods

An 8‐item survey was distributed among the members of the *German Testicular Cancer Study Group* (GTCSG) including urologists and oncologists in all German university hospitals between September 2020 and December 2020 (Supp. Figure 1). The questions focused on the imaging-based measurement of retroperitoneal lymph nodes for the classification of the initial clinical stage of GCTs in patients with retroperitoneal lymph-node metastasis and the assessment of a residual mass after chemotherapy. The imaging procedures and their interpretations were questioned in detail.

Statistical analysis was performed using the IBM SPSS Statistics system for Windows (v24.0) (Armonk, NY, USA). Categorical variables are presented as n (%). The data were analyzed using Pearson’s chi-square test. Differences were considered significant at *p* < 0.05.

## Results

The response rate was 93% (50/54); reasons for non-response were unknown. Of the 50 respondents, 96% worked in a hospital, 84% were specialized urologists (*n* = 42), and 16% (*n* = 8) were oncologists. All institutions performed > 10 imaging procedures for staging/restaging of GCT patients per year. Furthermore, > 30 imaging procedures per year were performed by 85.7% of the oncologists and 54.8% of the urologists (*p* = 0.123). All oncologists performed chemotherapy, while in the group of urologists, 92.8% performed chemotherapy (*n* = 39), and 97.6% performed retroperitoneal lymph-node dissection (*n* = 41).

**Fig. 2:**
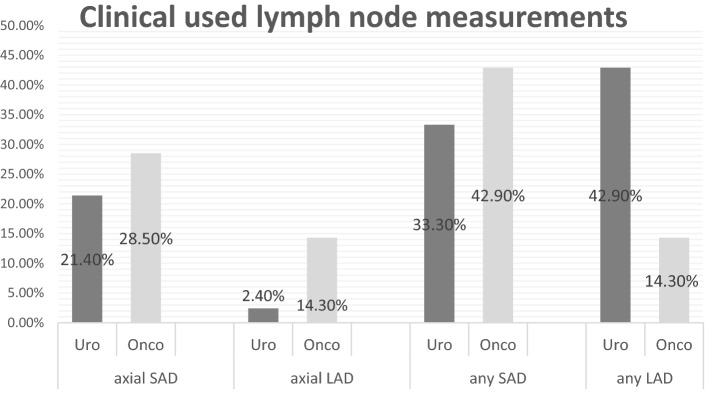
Clinical used lymph-node measurements. The analyzed diameters were separated in dimension [short- and long-axis diameter (SAD/LAD)] and plane [axial and not further defined (any) plane] for both urologists (Uro) and oncologists (Onco). The practice patterns of measuring the diameters did not differ between urologists and oncologists (*p* = 0.303)

There was an obvious inconsistency regarding the assessment of lymph-node size between the different medical disciplines. In the group of urologists, 54.7% assessed the lymph nodes depending on the short-axis diameter (SAD) (33.3% in any plane, 21.4% in the axial plane), while 45.3% assessed them according to the long-axis diameter (LAD) (42.9% in any plane, 2.4% in the axial plane, Fig. 2). In the group of oncologists, the assessment of lymph-node size mainly depended on the SAD (71.4%). In detail, 42.9% of oncologists assessed the SAD in any plane, while 28.5% measured it in the axial plane. Only 28.6% of oncologists considered the LAD (14.3% in any plane, 14.3% in the axial plane). The practice patterns of measuring lymph-node size did not differ between urologists and oncologists (*p* = 0.303). Additionally, only 17% of urologists (*n* = 7) included calculated lymph-node volume in their assessment of the initial clinical stage or residual mass after chemotherapy; however, none of the oncologists included such data (*p* = 0.224).

Additionally, we assessed the use of abdominal MRI instead of CT for initial clinical staging and follow-up imaging in patients with GCT. In the initial assessment, none of the oncologists and 11.9% of the urologists (*n* = 5) declared that they always performed MRI. Furthermore, 62.5% (*n* = 5) of oncologists and 50% (*n* = 21) of urologists performed MRI depending on individual patient factors and availability (*p* = 0.561). For follow-up imaging, the frequency shifted to an increased use of MRI in both groups. A total of 37.5% of oncologists and 31% of urologists always performed MRI for follow-up imaging, while only 12.5% of oncologists and 7.1% of urologists never used MRI (*p* = 0.783).

**Fig. 3:**
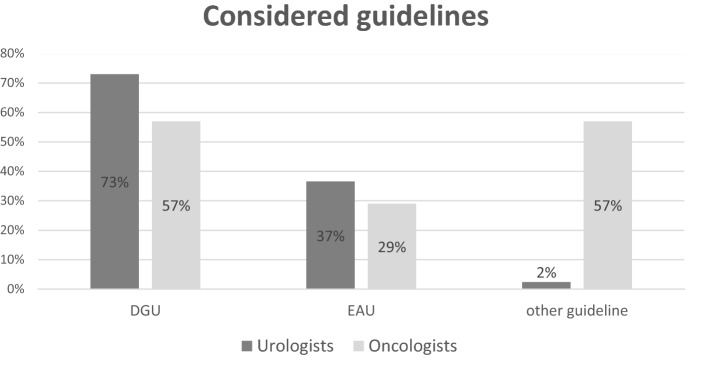
Considered guidelines. In the group of urologists, most specialists considered the guidelines of the German Society of Urology (DGU), while in the group of oncologists, other guidelines were often considered (e.g., *onkopedia*). More than one answer was possible

Furthermore, we analyzed the considered guidelines: While most urologists mentioned following the guidelines of the *German Society of Urology* (DGU, 73%, *n* = 30), most oncologists mentioned following another guideline, e.g., *onkopedia* (57%, *n* = 4, *p* < 0.001, Fig. 3). The EAU guidelines were used less frequently in both groups. This finding is of clinical relevance, as patients with the same disease might receive a different diagnostic imaging or therapy if institutions follow different treating guidelines.

## Discussion

This study was motivated by the interest in identifying practice patterns for measuring retroperitoneal lymph-node metastasis to assess the initial clinical stage and residual tumor mass after chemotherapy in patients with GCTs. None of the most common guidelines (EAU, NCCN, DGU, *onkopedia*) provide precise instructions on which plane of sectional imaging or which dimension (SAD/LAD) of the retroperitoneal lymph nodes should be assessed. According to the clinical Tumor/Node/Metastasis (cTNM) classification, the “greatest dimension” [15] should be considered. The EAU guidelines say, “size of metastases should be described in three dimensions, or at least by the greatest axial dimension” [11]. However, as we assessed most urologists and oncologists follow the German national guideline (DGU) and *onkopedia* which do not provide a clear statement of how to measure retroperitoneal lymph-node metastasis; likewise, the American national guideline by NCCN does not.

Therefore, we aimed to assess the practice patterns of German specialists frequently treating testicular cancer for measuring retroperitoneal lymph-node size in GCT patients. We found tremendous inconsistency among the experienced German centers in the assessment of retroperitoneal lymph-node metastasis and residual tumor mass after chemotherapy in patients with GCTs. Most urological and oncological specialists assessed their patients based on SAD in different radiological planes, which contradicts the actual instructions in guidelines from countries around the world. No significant difference was found between the assessments by urological and oncological specialists. This huge diagnostic variety can potentially result in under- or over-treatment of mostly young patients, with unnecessary higher recurrence rates or unnecessary acute and long-term therapeutic toxicity. However, in some GCT centers, measurements from radiologic reports are used for clinical treatment decision-making. Furthermore, the inclusion of volumetric assessment in our study group was low; only 17% of urologists, and no oncologists, included calculated lymph-node volume in their assessment.

Most actual guidelines refer to Leibovitch et al., who suggested a dynamic scale depending on the predicted landing zone of lymph-node metastasis according to the primary tumor site. Their data were based on a multivariate logistic regression of axial SAD which goes along with the current radiological guideline recommendations [16].

Cotner et al. [17] suggested that referencing the axial SAD instead of the previously used axial [16] LAD could reduce unnecessary post-chemotherapy retroperitoneal lymph-node dissection in patients with metastatic nonseminomatous GCTs. This contradicts the recommendations in the current clinical guidelines and refers to the radiological *Response Evaluation Criteria in Solid Tumors* (RECIST) 1.1 criteria, which is universally accepted in response assessments of most solid tumors and recommends “at baseline and in follow-up, only the short axis will be measured and followed” [18, 19]. Although RECIST 1.1 not only refers to the measurement of lymph-node size but also defines rules to categories lymph nodes in pathologic or non-pathologic and either target or non-target lesions. Furthermore, even if clear measurement instructions are included in the guidelines, some studies have proposed that guideline adherence, especially in centers treating testicular cancer, may be a common problem, particularly concerning inappropriate imaging [unnecessary positron emission tomography (PET) scans or brain or bone imaging] and overtreatment [20, 21]. This is consistent with our finding that German specialists treating GCTs follow different guidelines. Therefore, clear measurement instructions need to be published in all guidelines to reach all specialists.

Especially concerning measurement of the residual mass after chemotherapy, one study has suggested that volumetric assessments are a promising instrument to measure clinical response in patients with metastatic nonseminomatous GCTs treated with chemotherapy [17]. However, this has not yet been validated in a large group of patients. Limitations of lymph-node volumetry are measurement variabilities due to different software solutions resulting in a high interreader variability. Additionally, its use is time-consuming which hampers the clinical implementation. However, with the advent of artificial intelligence in clinical routine imaging, this picture might change and volumetric measurements could become more user-friendly [22, 23].

Furthermore, we found that in the setting of follow-up imaging, MRI was used by over 30% of urologists and oncologists. This is not only important for patients with contraindications to CT imaging, such as chronic kidney disease or allergies to radiocontrast agents, but also to reduce radiation exposure and the risk of developing secondary malignancies in the mainly young patients [24, 25]. Recent prospective studies suggest an equal detection rate of retroperitoneal lymph-node metastasis with MRI as with CT for experienced radiologists [13, 14, 26]. Because MRIs are still not as widely available as CTs and cost more time and money, widespread use for initial staging and follow-up may still take some time. A limitation of this study was the limited cohort of questioned urologists and oncologists. However, these institutions were highly selected as they are specialized in the treatment of GCT patients in Germany and we would have expected them to define clinical staging similarly. Within the institutions, there was always a single respondent who answered for the institution. Usually, the respondent was responsible in the clinic for the treatment of testicular cancer. We assume that each institution has their own practice guidelines how to diagnose and treat testicular cancer patients and/or treatment decisions are made in an interdisciplinary tumor board, resulting in a consistent procedure within an individual department. Another limitation might be that our questionnaire did not address radiologists but the evaluation how RECIST 1.1 is established in radiological workflow would need a separate comprehensive survey of radiologists in cancer centers, hospitals, and private practices which was beyond the purpose of this study. The aim of our study was to evaluate which of the described imaging-based diameters (SAD/LAD) is used by clinicians (urologists/oncologists) to define clinical stage. Furthermore, malignancy criteria for lymph nodes not only depend on size as small lymph nodes might harbor microscopic cancer infiltration and larger lymph nodes might be related to inflammatory processes. Therefore, the recent published lymph-node reporting and data system (Node-RADS) includes assessment of simple morphological characteristics and further improvements might be feasible within deep radiomics analysis [10, 27]. First promising results were shown in a study by Baessler et al. that examined lymph-node metastases in CTs prior to chemotherapy retroperitoneal lymph-node dissection [28]. The radiomics classifier yielded a significantly more accurate distinction than volumetry.

## Conclusions

There is no consensus in how to determine retroperitoneal lymph-node size in GCT patients for the initial clinical staging and assessments of residual mass after chemotherapy among specialized German institutions. We assume that this could be a problem present also in other countries, at the international level, since no common and shared international clinical guidelines contain precise information on correct lymph-node measurement in testicular cancer. At the European level, some EU policies are stimulating and encouraging international collaborations between experts connected in treatment of rare and complex urological conditions, including rare cancers such as testicular cancer, to establish shared and detailed guidelines [29, 30]. Moreover, further research is urgently needed to implement at least at the national-level clear recommendations for the “best” lymph-node measurement methods to avoid unnecessary, over- or under-treatment of testicular cancer, with adverse clinical consequences. Finally, our data seem to question the accuracy of clinical studies on patients with metastasized GCTs, especially regarding the reliability of reported comparisons of diseases with potential inaccurate clinical staging.

## Supplementary Information

Below is the link to the electronic supplementary material.

Supplementary file1 (DOCX 52 KB)

## Data Availability

The data sets generated and analyzed during the current study are available from the corresponding author on reasonable request.
