# Supplementary material for: German specialists treating testicular cancer follow different guidelines with resulting inconsistency in assessment of retroperitoneal lymph-node metastasis: clinical implications and possible corrective measures
Source: World J Urol. 2023 Apr 4;41(5):1353–8. doi: 10.1007/s00345-023-04364-5 (PMC10188422; doi:10.1007/s00345-023-04364-5)
Supplement: Supplementary file 1 — Supplementary file1 (DOCX 52 KB) [file 345_2023_4364_MOESM1_ESM.docx]

# Supplementary Figure 1

1. In which kind of **urooncological department** are you treating patients?
   - Medical practice External doctor with hospital affiliation
   - Hospital
2. How many **imaging examinations with CT/MRI** are performed in your department on average within one year for staging or restaging in patients with germ cell tumors of the testis?
   - 0 10-30
   - < 10 > 30
3. Which diagnostic criteria do **you assess in patients with retroperitoneal lymph node metastasis** due to testicular cancer?
   - Which diameter is relevant for therapy decisions?
     - **Short axis diameter in any dimension**

Or **just** in one plane:

- - - Transversal Coronal/Frontal
    - Sagittal
    - **Long axis diameter in any dimension**

Or **just** in one plane:

- - - Transversal Coronal/Frontal
    - Sagittal
  - Do you assess **volume** of lymph node metastasis?
    - Always Never
    - Sometimes: When? ___________________________________

1. Do you use **MRI for initial staging**?
   - - Always Never
     - Sometimes: When? ___________________________________
2. Do you use **MRI as follow-up imaging**?
   - - Always Never
     - Sometimes: When? ___________________________________
3. Do you perform **chemotherapy** in patients with testicular cancer?
   - - Yes No
4. Do you perform **retroperitoneal lymph node dissection** (pcRPLND) in patients with lymph node metastasis due to testicular cancer?
   - - Yes No
5. In accordance with which **guidelines** do you treat your patients?
   - - DGU (S3) EAU None/Others
